# The Systolic Anterior Motion After Mitral Valve Repair: A Narrative Review of Current Therapeutic Algorithms in the Operating Room

**DOI:** 10.1002/hsr2.72265

**Published:** 2026-09-17

**Authors:** Carmen Elena Opris, Horatiu Suciu, Catalin‐Constantin Badiu, Cosmin Ioan Opris, Alina Gabriela Negru, Simona Gurzu

**Affiliations:** ^1^ Department of Pathology George Emil Palade University of Medicine, Pharmacy, Science and Technology Targu Mures Romania; ^2^ Department of Adult and Children Cardiovascular Recovery Emergency Institute for Cardio‐Vascular Diseases and Transplantation Targu Mures Romania; ^3^ Department of Surgery George Emil Palade University of Medicine, Pharmacy, Science and Technology Targu Mures Romania; ^4^ Romanian Academy of Medical Sciences Bucharest Romania; ^5^ Department of Cardiovascular Surgery Emergency University Hospital Bucharest Romania; ^6^ Department of Cardiology and Cardiovascular Surgery Carol Davila University of Medicine and Pharmacy Bucharest Romania; ^7^ Department of Cardiology II Victor Babes University of Medicine and Pharmacy Timisoara Romania; ^8^ Research Center for Oncopathology and Translational Medicine (CCOMT) George Emil Palade University of Medicine, Pharmacy, Science and Technology Targu Mures Romania

**Keywords:** post‐operative, surgical mitral valve repair, surgical technique, systolic anterior motion

## Abstract

**Background and Aims:**

Systolic anterior motion (SAM) represents the displacement of the anterior mitral leaflet during ventricular systole toward the interventricular septum, causing an obstruction of the outflow tract. Most of the published data regards the primary SAM that is associated with hypertrophic cardiomyopathy. This review was focused on the management of SAM that occurs after mitral valve repair.

**Methods:**

To perform an update about the SAM occurring after mitral valve repair, data published in the last 15 years (2010–2025) were revised.

**Results:**

Out of the 354 publications that deal with SAM, 97 representative original papers matched our inclusion criteria: papers about the postoperative SAM and the description of SAM treatment. Although SAM can be successfully identified postoperatively, the paper highlights the importance of transesophageal echocardiography, in the operating room, as a diagnostic tool. The review also evaluates the treatment approaches, from conservative to surgical methods, and present them using representative graphs. It was showed that, postoperatively, conservative treatment is an option for patients with “negative SAM tolerance test.” When necessary, the type of surgical intervention should take into consideration the particularities of the mitral ring, leaflets, subvalvular apparatus, the mitral coaptation line, and the pre‐existing diseases.

**Conclusion:**

For proper management of post‐operative SAM, the implementation of an in‐house treatment algorithm should be established before the patient leaves the operating room.

## 1 Introduction

Systolic anterior motion (SAM) is defined as the displacement of the anterior mitral leaflet (AML) during ventricular systole toward the interventricular septum, causing an obstruction of the outflow tract. It is primarily associated with hypertrophic cardiomyopathy but also occurs in various contexts, such as general anesthesia or after mitral valve repair [1, 2, 3].

The incidence of SAM in patients undergoing mitral valve repair is 4% to 5% and rises to 14% in patients with a redundant myxomatous anterior leaflet [2, 3]. If related to surgery, SAM can be identified postoperatively, by transesophageal echocardiography, but sometimes is diagnosed only later after surgery [1, 2].

A late diagnosis of this postoperative complication, which is a serious cause of hemodynamic instability, might impair the result of mitral valve repair, sometimes requiring surgical re‐intervention [4]. The early recognition of this condition is very important for the management of these patients, and a therapeutic algorithm is important to be used in any clinical department.

The purpose of this manuscript is to present a comprehensive review of the literature of the last 15 years (2010–2025), indexed in the PubMed and Scopus databases, regarding the occurrence of SAM after mitral valve repair and the therapeutic attitude that should be followed. Being focused on the surgery‐related SAM, the review integrates the underlying mechanisms and the echocardiographic diagnostic approach with the therapeutic options—conservative and surgical. The main aim is to delineate the management of SAM, from the complex mechanism to the best protocols used for optimization of the patient care.

## 2 Selection Method

Using the keywords “surgical mitral valve repair AND systolic anterior motion” in the PubMed and Scopus databases, we analyzed 354 articles relating to the appearance of SAM after mitral valve repair. The inclusion criteria were the occurrence of postoperative SAM and the description of SAM treatment, in the operating room, in papers published in the last 15 years (2010–2025), before and after updating of the guidelines of the European Society of Cardiology/European Association of Cardio‐Thoracic Surgery regarding the management of valvular heart diseases (ESC/EACTS Guidelines) [5].

Out of 263 publications identified in the PubMed database, 168 articles published between 2010 and 2025 were selected for further analysis. Of these, only 61 representative studies matched our inclusion criteria and were eligible for this review. Similarly, out of 305 articles from the Scopus database, 186 articles were published between 2010 and 2025, but only 36 matched the inclusion criteria and were selected for further analysis.

After excluding the cases where SAM was present before surgery or due to hypertrophic cardiomyopathy or other cardiovascular diseases (primary SAM), as well as articles in languages other than English and duplicates, 97 papers were further analyzed.

## 3 Mitral Valve Repair – An Overview

According to the 2021 ESC/EACTS Guidelines [5], mitral valve repair is the preferred surgical approach whenever feasible and durable. It is indicated in all symptomatic patients with severe mitral regurgitation (MR), and in asymptomatic patients with evidence of dysfunction of the left ventricle (LV) [LVEF ≤ 60% or LVESD ≥ 40 mm].

Repair may also be considered in asymptomatic patients with preserved LV function but new‐onset atrial fibrillation or pulmonary hypertension (> 50 mmHg) [5]. In these patients, surgery can also be used to prevent the severe consequences of chronic mitral regurgitation, such as the dilation and deterioration of left ventricular function, atrial fibrillation, and pulmonary hypertension [5]. Furthermore, mitral valve repair should be the first choice of treatment in selected patients with degenerative mitral regurgitation [6, 7, 8, 9]. The presence of a flail leaflet in an asymptomatic patient, associated with left atrial enlargement (LA ≥ 55 mm) or left ventricular remodeling (LVESD ≥ 40 mm), represents an additional consideration for mitral valve repair [5].

An extensive analysis of the Mitral Regurgitation International Database showed that mitral valve repair is superior to mitral valve replacement in terms of the early and late outcomes [6]. More than 50 years ago, Carpentier presented three important rules regarding the mitral valve repair, which are still applicable: achieving a large coaptation zone (5–8 mm), maintaining complete mobility of the leaflets, and remodeling the dilated native valve ring with a prosthetic ring [8, 10, 11, 12, 13]. Neochordae implantation is an alternative to the techniques of cusp resection and native chordae reimplantation described by Carpentier for the treatment of leaflet prolapse treatment [14].

Whenever mitral valve repair is considered, a systematic evaluation pathway should be followed to maximize surgical success and minimize complications. This evaluation should include both general echocardiographic measurements and an assessment of the SAM risk factors, but also intraoperative surgical observations, such as identification of a myxomatous valve in addition to ventricular size and contractility, as well as mitral ring calcifications [9, 15, 16]. Nevertheless, several complications may occur immediately after mitral valve repair. Besides SAM, perforation, restriction of valve movement, residual prolapse, rupture of the left ventricle or left atrium, and coronary lesions, especially of the circumflex artery, due to its proximity to the posterior mitral ring, can occur [6, 9, 15, 16].

## 4 Mechanism of SAM

The exact mechanism of SAM has not yet been elucidated, although several theories have been suggested. It is believed that SAM occurs as the result of a Venturi phenomenon: the velocity of the blood flow, due to a thick interventricular septum that narrows the left ventricular outflow tract (LVOT), and drags the ventricular surface of the mitral valve anterior leaflet anteriorly, causing a gradient in LVOT and mitral regurgitation [17].

In a study conducted by Miura et al. regarding the occurrence of SAM after mitral valve repair, eight (7.7%) out of 104 patients were diagnosed with severe recurrent mitral regurgitation. The authors described a correlation between the presence of the sigmoid‐looking interventricular septum and the anterior mitral leaflet (AML) reaching the septum, as well as the presence of postoperative SAM. Four patients required re‐intervention [18]. Similar observations were noted by Varghese et al., who studied a group of 375 patients, of whom 8% suffered from SAM [19]. In an analysis performed by Ashikhima et al., there was no significant association between the thickness of the basal septum and the risk of SAM, as SAM might be observed in the absence of asymmetric septal hypertrophy [20]. In conclusion, the Venturi phenomenon does not entirely explain the presence of SAM.

Both hypovolemia, by accentuating the Venturi phenomenon in LVOT, and hypervolemia, through pulmonary overload and straight cavities with interventricular septum (IVS) pushing toward the left ventricle (LV) and narrowing of the LVOT, can cause SAM after mitral valve repair [4, 17]. Depending on the degree of anterior movement, the severity of SAM can vary leading from minor mitral dysfunction to severe regurgitation and outflow tract obstruction, thus presenting with hemodynamic instability [21, 22].

Tachyarrhythmias can induce SAM by raising the heart rate, decreasing the diastolic time, and reducing ventricular filling. Positive inotropes increase ventricular contractility, resulting in a narrowing LVOT, thus increasing the severity of SAM [4]. Postoperative LV dyssynchrony is another cause of postoperative SAM and mitral regurgitation [23].

The surgical techniques that are utilized for mitral valve repair may also induce SAM. Using a small, rigid, or complete annuloplasty induces SAM by disrupting mitral annular and aortic root dynamics [24, 25, 26]. In addition, a disbalance between the anterior and posterior leaflet height as well as between the leaflet area and mitral valve orifice may be a cause of SAM [24]. If the coaptation line is disposed anteriorly after mitral valve repair, SAM may appear. Other operative reasons for SAM include inadequate posterior leaflet height reduction or overly long neochordae [21, 27]. Redundant chordae tendineae may protrude in the LVOT in early systole, producing SAM [28]. The intrinsic anomalies of the mitral apparatus, for example, a myxomatous mitral valve with excess leaflet tissue, also make an important contribution to SAM occurrence.

## 5 Diagnosis of SAM in the Operating Room ‐ Intraoperative Transesophageal Echocardiography

The most important parameters considered in mitral valve repair are the echocardiographic measurement of A2 and P2 for reconstructing the normal 2:1 ratio of anterior to posterior leaflet lengths and choosing the ring size based on the A2 length. The purpose is to achieve a good coaptation zone (5–10 mm) without SAM. This method is applicable assuming no residual elongation or restriction of the mitral valve chordae are present and that the measurements are accurate [29].

Pre‐operative echocardiographic assessment is important for identifying risk factors for the occurrence of SAM after mitral valve repair. Left ventricular end‐diastolic dimension (LVEDD) < 45 mm, basal‐interventricular septal diameter in diastole > 15 mm, aorto‐mitral angle < 120°, posterior leaflet height > 15 mm, coaptation‐septal distance (c‐sept) < 25 mm, asymmetry of A2: anterolateral A2 height‐posteromedial A2 height ≥ 5 mm, anterior/posterior heights leaflet ratio < 1,3, the bileaflet prolaps and a small, hyperkinetic heart are the risk factors for SAM that can be identified pre‐operative [19, 20, 30, 31, 32, 33].

The importance of postoperative transesophageal echocardiography, before leaving the operating room, is crucial for evaluating the result of mitral valve repair. Ideally, it should be performed after weaning the patient from cardiopulmonary bypass but before closing the chest. A good mitral valve repair result means a maximum minor mitral regurgitation (0 or maximum 1+ regurgitation). A very important issue that must be considered is the hemodynamic status of the patient immediately after cardiopulmonary bypass weaning. For an accurate result, the blood pressure must be at least close to normal. Furthermore, one must wait for the contractility of the left ventricle to recover after bypass [33]. The measurement of the LVOT gradient using the transgastric, long‐axis view has a major importance for the diagnosis of SAM. With the probe advanced in the patient's stomach, in extreme anteflexion, and by increasing the array angle in a vertical plane, the LVOT is visualized, and the gradient can be measured accurately.

## 6 Treatment of Postoperative SAM

### 6.1 Conservative Treatment of SAM

Considering that both hypovolemia and hypervolemia can influence SAM, a proper hemodynamic status when weaning from aortocoronary bypass is necessary [21]. Therefore, minor dysfunction can be mostly corrected with volume administration, increasing preload and afterload, concomitant to beta‐adrenoceptor blockade [34, 35, 36, 37]. Conservative measures used to treat transient intraoperative SAM do not improve the late outcome after mitral valve repair [38, 39]. In contrast, severe forms require surgical correction. The intraoperative management should be conducted based on the characteristics of the mitral valve complex and the shape of the LV, as well as the risk of reintervention [40].

**FIGURE 1:**
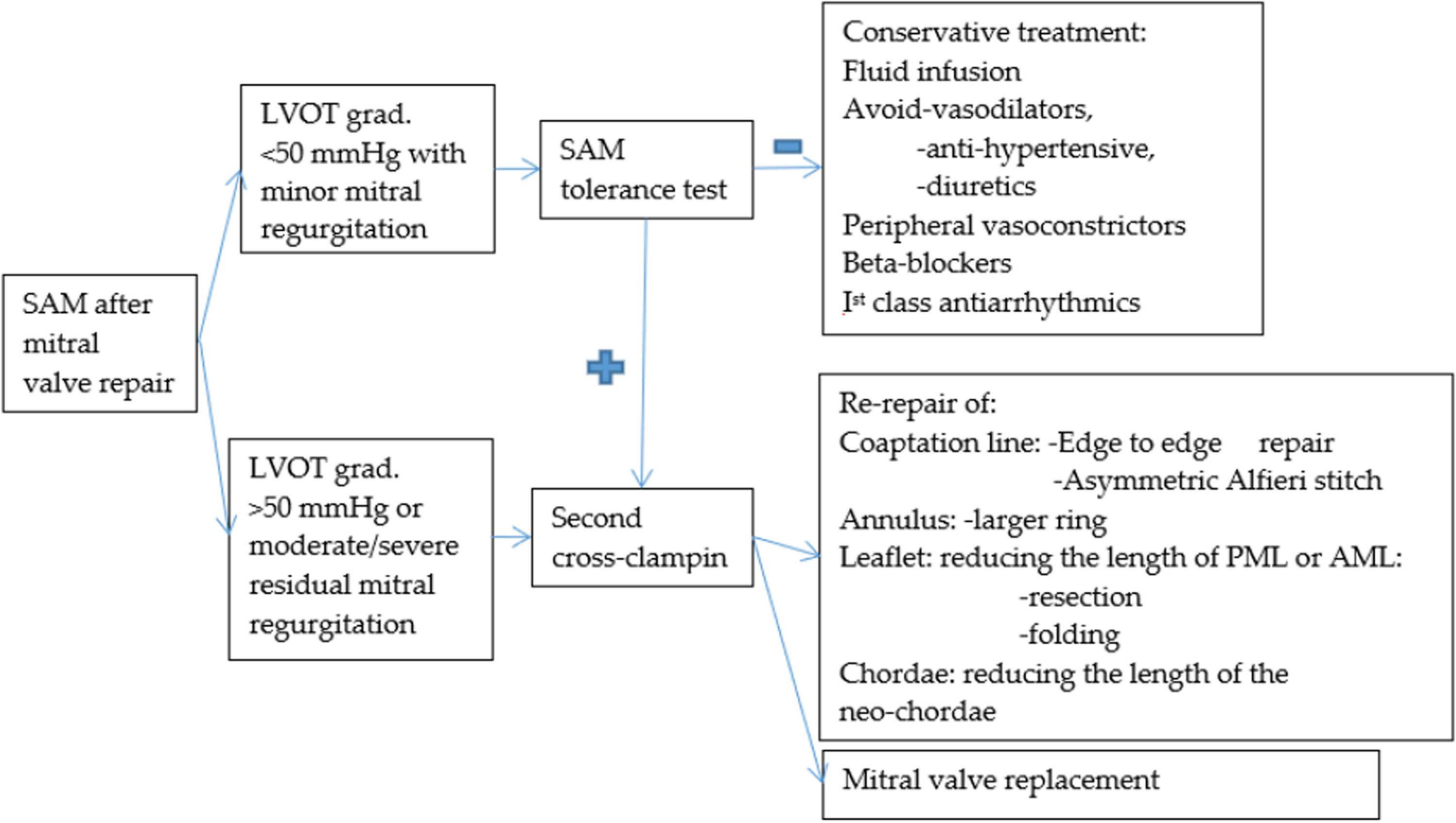
Treatment algorithm for immediate postoperative SAM is based on the value of the left ventricular outflow tract (LVOT) gradient. Conservative versus surgical treatment is decided in the operative room by the SAM tolerance test [34, 35, 36, 37, 38, 39, 40, 41, 42, 43].

If SAM is present after weaning from bypass, a “SAM tolerance test” might help the surgeon for the best intraoperative decision. This test creates hemodynamic conditions to aggravate the SAM dysfunction, in the operating room, through the administration of vasodilators and positive inotropes, or by increasing the cardiac frequency. If the SAM dysfunction does not aggravate, Manecke et al. advocate that the postoperative vasodilation, hypovolemia, and tachycardia will not deteriorate the hemodynamic status, and the patient may be discharged with conservative treatment [41] (Figure 1).

To avoid SAM, Varghese et al. also proposed a management algorithm. First, the ring chosen for annuloplasty must be the right size. Furthermore, if the posterior mitral leaflet height is more than 15 mm above the coaptation line, resection of the leaflet is recommended. Transesophageal echocardiography is mandatory before cardiopulmonary bypass weaning. Hence, a LVOT gradient above 50 mmHg requires immediate surgical resolution. On the other hand, if the mitral valve regurgitation is minor and the gradient is less than 50 mmHg, drug treatment is preferred. This consists of volume administration and subsequent beta blocker medication, in addition to avoiding antihypertensives and diuretics, while maintaining the systolic blood pressure over 135 mmHg [42] (Figure 1).

Positive inotropes such as epinephrine increase the ventricular contractility, which results in a narrowing LVOT, thus increasing the severity of SAM, and should be used with caution. It is also recommended to avoid the peripheral vasodilators. Treatment should include the discontinuation of positive inotropes and fluid infusion [43] (Figure 1). For the treatment of tachyarrhythmias, beta blockers are useful when their administration is not contraindicated [15, 43].

With a plasma half‐life of 4 min, landiolol is a cardioselective ultra‐short‐acting b1‐adrenergic receptor blocking agent that can attenuate the SAM of the mitral valve after mitral valve annuloplasty [15]. It is preferable to use beta blockers in continuous injection in comparison to boluses, which have a more aggressive effect on lowering blood pressure. Postoperatively, in case of beta blocker resistance, oral disopyramide up‐titrated to the maximal tolerated dose (300–750 mg) administered in divided doses two or three times daily might be a solution [44]. Cibenzoline is another first‐class anti‐arrhythmic agent that may reduce SAM in the early postoperative period by decreasing the left ventricular contractility. It is indicated especially when other conservative measures fail to reduce postoperative SAM or are contraindicated and is recommended for use before deciding on surgical repair [45].

### 6.2 Surgical Treatment of SAM

Depending on the etiology of SAM after mitral valve repair, the surgical armamentarium includes different resection or plication techniques regarding the elongated leaflet, annuloplasty ring replacement with a larger‐sized ring, and adequate adjustment of the neochordae length.

If an immediate reintervention is necessary for SAM after mitral valve repair, mitral valve replacement should be the first solution in patients with an increased reintervention risk or those who have had a prolonged clamping time [46]. A second cross‐clamping may be necessary to make these adjustments, but if the cross‐clamping time is short, the outcome should not be influenced [47, 48].

#### 6.2.1 Surgical Techniques Involving the Mitral Ring

A surgery‐related reason for SAM is the use of a small artificial ring, as the entire mitral coaptation line might be displaced anteriorly [24, 25, 26]. In patients at a high risk of post‐valve repair SAM, the usage of a larger ring is required [49, 50]. Varghese et al. utilized a mean ring size of 32.2 +/−4 mm; 8% of the 375 patients developed SAM after mitral valve repair, with the ring size causing no differences between the two groups (with or without postoperative SAM) [19].

**FIGURE 2:**
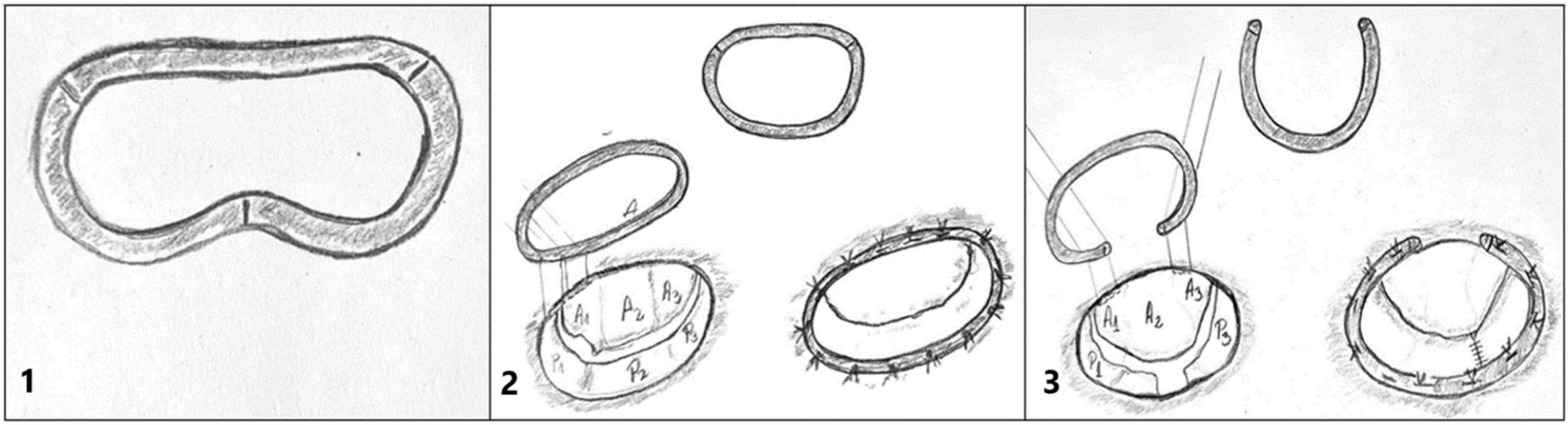
Examples of rings used for mitral annuloplasty: 1. Rigid complete ring; 2. Semi‐rigid complete ring; 3. Flexible incomplete ring.

The use of a complete rigid ring, particularly an overly small one, determines the appearance of SAM due to the disruption of mitral annular and aortic root dynamics [14, 49, 50, 51, 52, 53]. An open rigid ring offers satisfactory long‐term results [52]. The size of the annuloplasty ring may be determined by using different techniques. More than 40 annuloplasty rings with different shapes and many annular sizers are available (Figure 2), but, as Bothe et al. suggested, the current strategies for sizing annuloplasty rings are still contradictory [53]. They observed that although ring dimensions are identical, the dimensions of the sizers from different manufacturers differ, and sometimes the dimension of the sizer does not match those of their annuloplasty ring. Therefore, the unification of annuloplasty ring, band, and sizer labeling could be an initial step in simplifying the approach to ring sizing [53, 54, 55]. A new technique proposed by Okamoto et al. consists of setting commissures or trigones at 1/4 of the annular circumference instead of 1/3, as current commissure and trigone‐based sizing strategies of the manufacturers recommend, when measuring the AML [56].

Some authors recommend the use of a partial ring, which might prevent the occurrence of SAM, but this hypothesis has not been clearly demonstrated. It might be a solution for patients with myxomatous valves, but not for those with functional mitral regurgitation. In the latter form, when the leaflets are structurally normal, not redundant, the use of a complete ring seems to improve freedom from recurrent mitral regurgitation [57, 58].

Another satisfactory solution that is also cost‐effective was described by Pitsis et al [59]. They suggested that using a personalized ring, constructed in the operating room out of a Dacron sheet and titanium ligating clips, might help to prevent SAM after mitral valve repair [59]. Based on the same idea, the use of an adjustable mitral ring in mitral valve repair, implanted under real‐time echocardiography guidance, might decrease the risk of SAM [60, 61, 62].

Micro‐sizing the mitral annulus after mitral annuloplasty confers adjustment of the size of the mitral annulus. It is supposed to prevent residual leaks, mitral stenosis, or SAM after the mitral valve repair [59]. For a better adjustment of the annuloplasty ring, Seki et al. suggested a thermally deformable ring, made of polycaprolactone, that is suitable for asymmetric repair of the mitral ring by deforming the ring using intraoperative heating [63].

In another experimental study performed on domestic pigs, the authors implanted a new annuloplasty system, consisting of two half‐rings with a saddle‐shaped outline. They concluded that the three‐dimensional dynamics of the mitral ring were maintained and the annulus restraining forces were reduced, leading to an increased repair durability [49, 64, 65]. Han et al. demonstrated in their analysis with 78 patients that the use of a saddle ring along with artificial chordae transplantation in degenerative mitral valve repair has excellent midterm results [66].

#### 6.2.2 Surgical Techniques Involving the Mitral Leaflets

In a study conducted by Manabe et al. on 179 patients with mitral valve repair, 15 patients developed SAM. The authors concluded that the change in mitral valve morphology after valve repair, with the coaptation point shifted toward the interventricular septum and the amount of excessive anterior leaflet that extends beyond the coaptation point, are important factors in SAM development. Even if the anatomy of the native mitral valve is important for predicting SAM, no differences in the preoperative mitral valve morphologies between the SAM and non‐SAM groups were found. However, a smaller ring and, therefore, a larger AML was described in the SAM group versus the non‐SAM group [24, 25].

**FIGURE 3:**
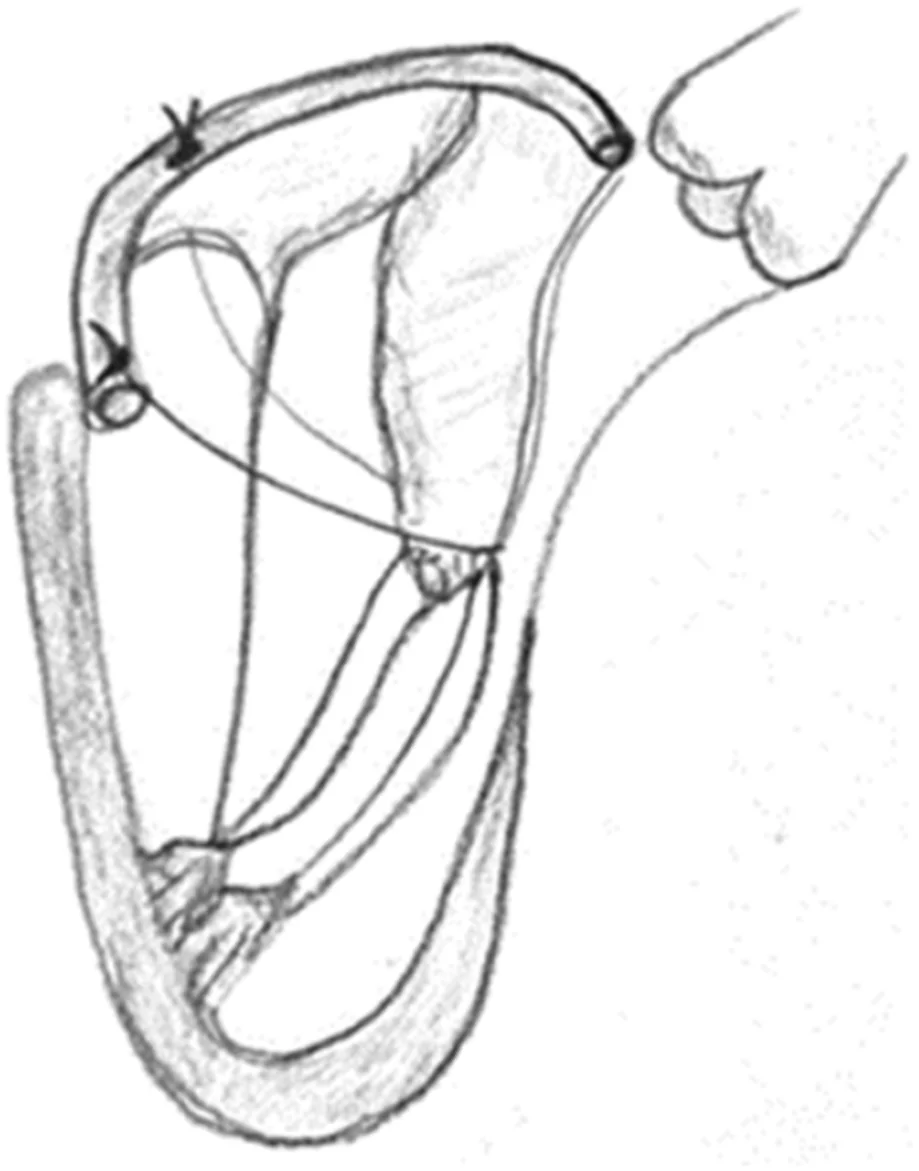
Surgical repair of mitral valve using the mitral leaflets. Paradoxical stitches aim to discipline the anterior leaflet to avoid postplasty systolic anterior motion. Picture adapted with permission after Kassem S et al. [67, 68].

An inadequate posterior leaflet height reduction may also lead to SAM after mitral valve repair [21, 26]. There are two equally important strategies for a good repair technique and SAM prevention: “resect” versus “respect rather than resect.” Previously used techniques include posterior mitral leaflet (PML) resection for moving the valvular coaptation line posteriorly, as well as AML resection to reduce the amount of excessive leaflet tissue. The “respect rather than resect” technique involving the anterior leaflet recommends fixing it in a way that makes entering in the LVOT during systole no longer possible. The procedure described by Kassem et al. utilizes two artificial chords implanted in the posterior and anterior papillary muscles, then anchored to the mid‐anterior mitral annulus to keep the anterior leaflet away from the septum during late diastole [67, 68, 69, 70, 71] (Figure 3).

In case of posterior mitral valve prolapse, Woo et al. described a non‐resection leaflet remodeling technique that efficiently utilizes a single Gore‐Tex suture to posteriorly anchor and support, while remodeling the posterior leaflet [72, 73].

**FIGURE 4:**
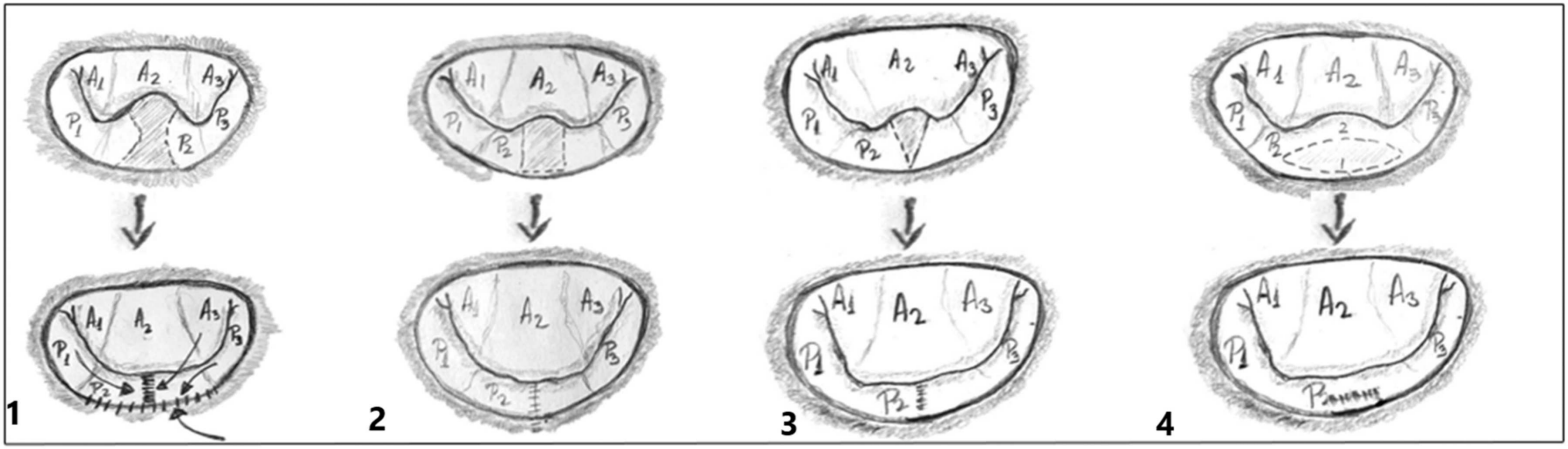
Surgical repair of mitral valve, using the mitral leaflets. The length of the posterior mitral valve may be reduced by different types of valvular resection: 1. Hourglass‐shaped resection ‐ adapted with permission after Sawazaki and all [75]; 2. Quadrangular resection; 3. Triangular resection; 4. Parannular elliptical posterior resection ‐ adapted with permission after Da Col U and all [76].

On the other hand, the length of the posterior mitral valve may be reduced by sliding (which includes resection) or folding. The resection area might have different shapes and sizes. Although the most frequent leaflet resections are triangular or quadrangular, other shapes may be used [74] (Figure 4).

The hourglass‐shaped resection technique, described by Sawazaki, rather than quadrangular resection, seems promising [75]. Using a butterfly shape resection, consisting of two triangular resections, one at the free margin and one at the base of the valve, can solve the problem of a posterior leaflet prolapse by reducing the excessive leaflet height and providing a good control of the coaptation extent [77, 78, 79]. The parannular elliptical resection of the PML base, described by Da Col U et al., prevents the distortion and the weakening of the mitral ring [76] (Figure 4).

**FIGURE 5:**
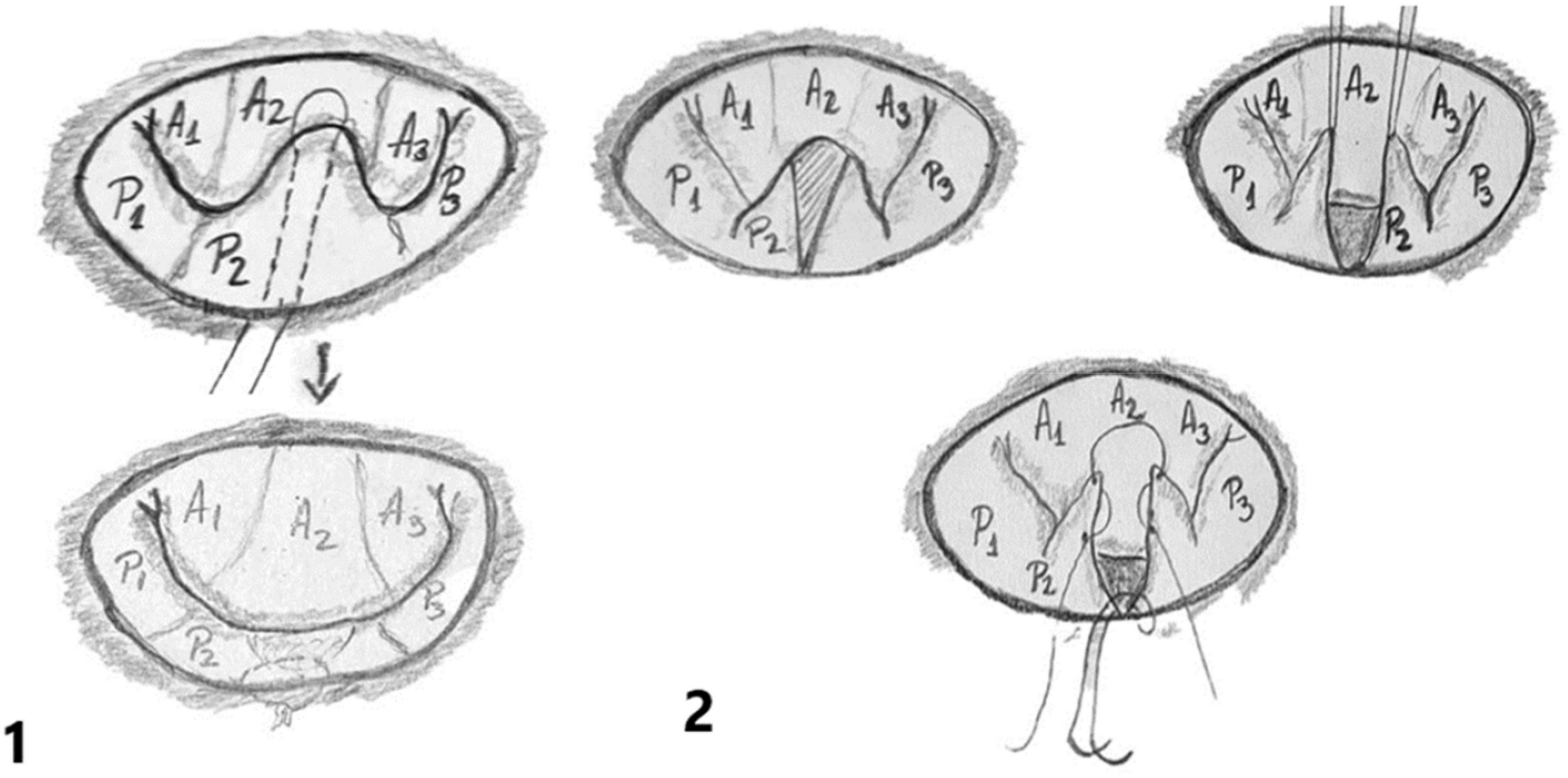
Surgical repair of mitral valve, using the mitral leaflets. 1. Foldoplasty consists of non‐resection folding of the posterior valve. Picture adapted with permission after Cevasco M et al. [80]; 2. Partial folodplasty ‐ adapted with permission after Abicht TO et al. [81].

While resection is irreversible, utilizing a folding technique for the posterior mitral valve, known as foldoplasty (Figure 5), gives the surgeon the opportunity to rearrange the valve repair, in case of unsatisfactory results [82]. Foldoplasty might prevent SAM with a satisfactory midterm outcome [80, 83]. Partial foldoplasty, described by Abicht et al., together with a complete ring chosen based on the A2 length, is another simple technique for reducing the posterior leaflet height by resection and then folding and establishing an A2/P2 ratio of 2:1 by measuring the intraoperative the lengths of the two leaflets using a custom caliper [81].

Manabe et al. consider that, in patients with a small hyperkinetic heart, suturing the free edges of the anterior and posterior leaflets together might avoid SAM. These edge‐to‐edge sutures do not narrow the mitral valve area but represent a simple method for preventing the anterior movement of the anterior leaflet during systole and thus avoiding the development of SAM [50, 84].

#### 6.2.3 Surgical Techniques Involving the Subvalvular Apparatus

The risk of SAM is also based on the chordae tendineae, papillary muscles, and also the mitral valve aging and genetic profile [16, 85, 86]. Overly long second‐order chordae represent a predictor for SAM [87]. Neochordae anchored on the left ventricular apex may narrow the LVOT, causing SAM [88, 89]. But, as the geometry of the left ventricle cavity and papillary muscle orientation may differ in different pathological situations, anchoring the neochordae on the tip of the corresponding papillary muscle does not apply in any pathology [89].

In 2017, the first case of a minimally invasive NeoChord implantation utilizing the transapical off‐pump approach to correct a posterior mitral leaflet prolapse under real‐time transesophageal guidance was reported. The result was the complete elimination of SAM by a beating heart, minimally invasive procedure [90].

Sometimes the strands used for mitral valve repair can be too long. Once the annuloplasty ring is implanted, it becomes very difficult to shorten the neochordae [14, 91]. One of the methods, used for shortening, was proposed by Alameddine et al. A new Gore‐Tex strand attached to the base of the first neochordae is woven on the existing neochordae, shortening it to the required length [92].

**FIGURE 6:**
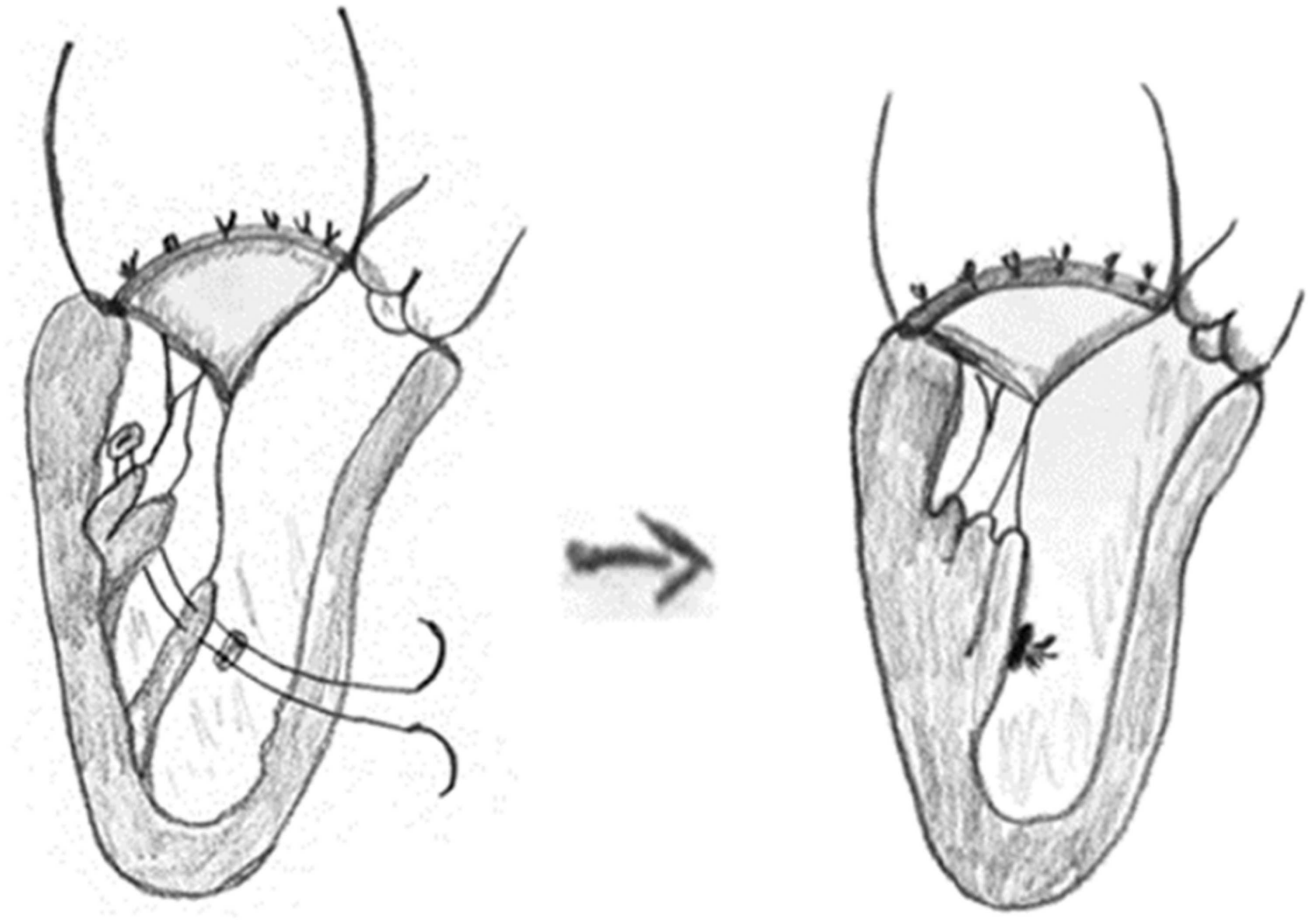
Surgical repair of mitral valve, in patients with papillary muscle displacement. The papillary muscles are displaced by the interventricular septum being brought closer to the posterior wall, and thus the LVOT is released. Picture adapted with permission after Sakaguchi et al. [21]. LVOT, left ventricular outflow tract.

Besides neochordae, papillary muscle displacement can also cause SAM and LVOT obstruction, even in the absence of hypertrophic cardiomyopathy, by causing slack and an increased residual length and laxity of the anterior leaflet [27, 68]. In such cases, Sakaguchi et al. demonstrated that papillary muscle reorientation can solve the situation. This method was previously described for the treatment of SAM occurring in hypertrophic cardiomyopathy [21] (Figure 6).

#### 6.2.4 Surgical Techniques Involving the Mitral Coaptation Line

**FIGURE 7:**
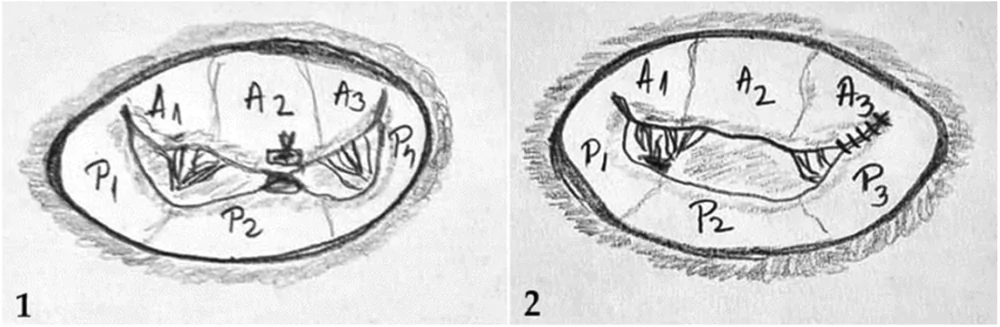
Surgical repair of mitral valve, using the mitral coaptation line: 1. Alfieri's edge‐to‐edge repair (classic procedure) [32, 33]; 2. Alfieri asymmetric stitch [2].

Sometimes Alfieri's edge‐to‐edge repair technique might be useful [32, 33, 87, 92, 93, 94, 95, 96]. However, this method does not prevent postoperative SAM when the obstruction is induced by the lateral segment (A1) of the anterior leaflet. In this situation, adapted techniques are indicated [2, 97]. Those proposed by Khalpey et al., refers to a partial anterior leaflet valve repair [2] (Figure 7).

## 7 Further Perspectives

Despite the progress of mitral valve repair techniques, SAM is a complication that should be considered when the patient becomes unstable after cardiopulmonary bypass weaning. Transesophageal echocardiography, in the operating room, has an important role in detecting and establishing the severity of the risk factors and postoperative SAM. Based on the data revealed by the echocardiographic examination, the cardiologist and cardiac surgeon need to decide the better algorithm of post‐operative SAM. When the SAM tolerance test is negative, conservative treatment might be implemented, but when necessary, the type of surgical intervention should take into consideration the particularities of the mitral ring, leaflets, subvalvular apparatus, the mitral coaptation line, and the pre‐existing diseases.

## Author Contributions

**Carmen Elena Opris:** project administration. **Carmen Elena Opris**, **Catalin‐Constantin Badiu**, and **Simona Gurzu:** conceptualization, original draft preparation. **Carmen Elena Opris**, **Horatiu Suciu**, **Cosmin Ioan Opris**, and **Alina Gabriela Negru:** investigation. **Catalin‐Constantin Badiu**, **Cosmin Ioan Opris**, and **Alina Gabriela Negru:** data curation. **Carmen Elena Opris**, **Horatiu Suciu**, and **Cosmin Ioan Opris:** methodology. **Horatiu Suciu**, **Catalin‐Constantin Badiu**, and **Cosmin Ioan Opris:** visualization. **Simona Gurzu**, **Alina Gabriela Negru**, and **Horatiu Suciu:** validation. **Catalin‐Constantin Badiu:** software. **Catalin‐Constantin Badiu**, **Cosmin Ioan Opris**, and **Alina Gabriela Negru:** formal analysis. **Simona Gurzu:** review, funding acquisition, resources, editing and supervision.

## Funding

The authors have nothing to report.

## Ethics Statement

This is a review of the literature. No Ethical Committee approval was necessary.

## Conflicts of Interest

The authors declare no conflicts of interest.

## Transparency Statement

The lead author, Carmen‐Elena Opris, affirms that this manuscript is an honest, accurate, and transparent account of the study being reported; that no important aspects of the study have been omitted; and that any discrepancies from the study as planned (and, if relevant, registered) have been explained.

## Data Availability

Carmen‐Elena Opris, the first author and manuscript guarantor, had full access to all of the data in this study and takes complete responsibility for the integrity of the data and the accuracy of the data analysis.
